# Microscale multicircuit brain stimulation: Achieving real-time brain state control for novel applications

**DOI:** 10.1016/j.crneur.2022.100071

**Published:** 2022-12-29

**Authors:** Yuri B. Saalmann, Sima Mofakham, Charles B. Mikell, Petar M. Djuric

**Affiliations:** a Department of Psychology, University of Wisconsin-Madison, Madison, WI, USA; b Wisconsin National Primate Research Center, University of Wisconsin-Madison, Madison, WI, USA; c Department of Neurological Surgery, Stony Brook University Hospital, Stony Brook, NY, USA; d Department of Electrical and Computer Engineering, Stony Brook University, Stony Brook, NY, USA

**Keywords:** Deep brain stimulation, Machine learning, Consciousness, Cognitive control, Neuromodulation

## Abstract

Neurological and psychiatric disorders typically result from dysfunction across multiple neural circuits. Most of these disorders lack a satisfactory neuromodulation treatment. However, deep brain stimulation (DBS) has been successful in a limited number of disorders; DBS typically targets one or two brain areas with single contacts on relatively large electrodes, allowing for only coarse modulation of circuit function. Because of the dysfunction in distributed neural circuits – each requiring fine, tailored modulation – that characterizes most neuropsychiatric disorders, this approach holds limited promise. To develop the next generation of neuromodulation therapies, we will have to achieve fine-grained, closed-loop control over multiple neural circuits. Recent work has demonstrated spatial and frequency selectivity using microstimulation with many small, closely-spaced contacts, mimicking endogenous neural dynamics. Using custom electrode design and stimulation parameters, it should be possible to achieve bidirectional control over behavioral outcomes, such as increasing or decreasing arousal during central thalamic stimulation. Here, we discuss one possible approach, which we term microscale multicircuit brain stimulation (MMBS). We discuss how machine learning leverages behavioral and neural data to find optimal stimulation parameters across multiple contacts, to drive the brain towards desired states associated with behavioral goals. We expound a mathematical framework for MMBS, where behavioral and neural responses adjust the model in real-time, allowing us to adjust stimulation in real-time. These technologies will be critical to the development of the next generation of neurostimulation therapies, which will allow us to treat problems like disorders of consciousness and cognition.

## Highlights

• Neurostimulation for brain diseases may need fine modulation of multiple circuits.
• Goal of microscale multicircuit brain stimulation is to normalize these circuits.
• Machine learning can be used to optimize the brain state in a closed-loop paradigm.
• Algorithm adjusts stimulation in real time based on behavioral and neural responses.

## Graphical abstract

**Figure:**
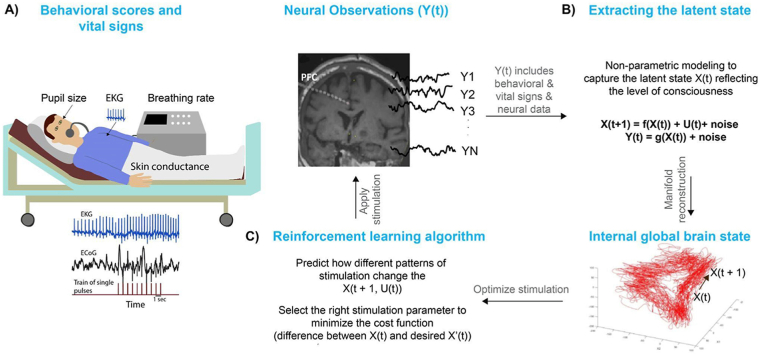

## 1 Introduction

Deep brain stimulation (DBS) is a treatment for Parkinson's disease, essential tremor, and epilepsy, consisting of electrical stimulation of a variety of brain targets (Karas et al., 2013). The net effects of DBS are complex and are related to the volume of tissue activated (VTA); VTA reflects the biophysical properties of the electrode and the geometry of the electrode contacts (Butson and McIntyre, 2005). For many years the only approved DBS system (Medtronic) had four ∼1.27 mm contacts, spaced either 0.5 or 1.5 mm apart. Newer systems have directional capabilities (Dembek et al., 2017). However, spatial resolution remains fixed, and only coarse modulation of brain function is possible with DBS.

In addition to limited spatial resolution, the effects of DBS are further complicated by a number of tissue-specific factors, including different cell types, different firing properties of local neurons, differing myelin content, and state/context-dependent activity changes. Target areas also have differing long-range connectivity patterns. If DBS approaches do not adequately consider these factors, outcomes may be suboptimal (Butson et al., 2007). Moreover, the effects of DBS on ongoing neural activity are incompletely understood. According to current models, DBS can excite, inhibit, or regularize activity, leading to impaired information transmission or an “information lesion” (Chiken and Nambu, 2016; Dorval and Grill, 2014). Taken together, these data support the view that, in most cases, DBS robustly downregulates pathological excitability of nuclei and/or large-scale oscillatory patterns, rather than necessarily restoring healthy neural activity. It is important to note that DBS robustly improves quality of life of patients with Parkinson's disease, essential tremor and dystonia. Nonetheless, because restoring healthy activity is a better goal than coarse modulation of excitability, we focus on this goal for the rest of the paper.

## 2 What spatial resolution is sufficient?

The structural and functional organization within and across brain areas is heterogeneous. Because of “cortical chauvinism” (the view that everything interesting happens in the cortex) and the difficulty of obtaining direct subcortical recordings, the internal organization of subcortical areas is often not fully taken into account. Nonetheless, applicable to both cortical and subcortical areas, there are neurons with different functional properties, which are often systematically organized within an individual area. For example, in motor cortex, there is evidence for motor map organization based on microstimulation experiments (Vitek et al., 1996). A second applicable model is the “action map,” in which clusters of neurons represent particular behaviors (Graziano, 2006). Moreover, the basal ganglia and subthalamic nucleus also show evidence of somatotopic organization (Rodriguez-Oroz et al., 2001). In light of the functional organization within each current DBS target area, independent manipulation of individual neurons does not seem necessary (nor possible) because of the similar response properties of nearby neurons, i.e., co-activation of nearby neurons under natural circumstances could be mimicked by stimulating the localized neuronal group. To specifically manipulate neurons encoding similar functional attributes, the stimulation influence should ideally match the spatial grain of functional organization within the individual brain area. That is, rather than a relatively large sphere or pear-shaped influence over an area(s), as in current VTA models (Butson et al., 2007; Butson and McIntyre, 2005), there should be focused stimulation onto the relevant functional organizational unit(s), which may need multiple foci in the case of distributed functional representations. Further, the focused stimulation may need to flexibly move across the functional topography/map in individual areas according to behavioral needs, and this spatiotemporal variation in applied current across electrodes (see section 4) may be achieved using microscale multicircuit brain stimulation (MMBS; see section 6). The precise scale of focused stimulation will depend on the internal functional organization of the target area, but generally speaking this would likely be equal to or less than 250–500 μm across, i.e., less than that typically achieved with current DBS protocols using macroelectrodes.

## 3 What spatial resolution is possible?

The currently available methods for manipulating neural activity up to high frequencies are electrical stimulation and optogenetics. While optogenetics offers cell-type specificity, there are drawbacks. First, optogenetics faces limits in spatiotemporally varying control, i.e., every new group of neurons to be independently controlled would need a new optical fiber, with its own size limits, and light source. Second, there is the potential for immunogenicity (Mendoza et al., 2017). Third, advances in rodents have not readily translated to primates (Servick, 2020). Finally, optogenetics currently uses repetitive, constant-frequency stimulation patterns, which do not resemble nor induce the dynamic activity patterns in the brain necessary for cognition. In comparison, electrical stimulation is currently applied as either DBS using one or few large electrode contacts (around 1 mm diameter) with one large VTA (>10 mm^3^; Butson et al., 2007; Dembek et al., 2017), orders of magnitude poorer than the necessary spatial resolution to independently control individual groups of neurons to enhance cognition; or highly localized microstimulation via one or few microelectrodes, which targets one group of neurons (Nichols and Newsome, 2002; Mazurek and Schieber, 2021) but not other groups distributed over large expanses of the brain which contribute to distributed cognitive computations. This latter issue, however, can be overcome by using a high number of microelectrodes spanning one or more brain areas, i.e., the basis of the MMBS approach, and our focus hereon.

Microstimulation of cortical and subcortical areas can modify both perception and action. To estimate the spatial resolution possible in such neuromodulation, we will discuss microstimulation experiments of the cerebral cortex in non-human primates (NHPs) using a single sharp electrode with an exposed tip (∼1–10 μm in size). We will then discuss thalamic microstimulation experiments, to show findings generalize to subcortical areas, and later discuss (section 6) the extension of these findings to humans, based on shared brain architecture.

The mammalian cerebral cortex is organized into columns, with diameters of ∼0.5 mm (Hubel and Wiesel, 1966). Neurons within a single column typically share response properties; for instance, neurons in a given column of the motion-sensitive middle temporal area (MT) share a preferred motion direction, and the preferred direction differs across columns (Shadlen and Newsome, 1996). Stimulation of MT neurons with known motion-direction preferences caused NHPs to report stimuli moving in the corresponding direction (Groh et al., 1997; Salzman and Newsome, 1994). Thus, microstimulation can drive behavior, in the appropriate context. Stimulating across multiple columns in MT can have complex effects; it may lead to a “winner-take-all” outcome when activating two columns with near opposing preferred directions, or vector-averaging when activating two columns with closer preferred directions (Nichols and Newsome, 2002). Thus, the topographic organization of brain areas can be exploited to achieve some predictability for cortical stimulation.

The situation is similar in subcortical regions. The lateral geniculate nucleus (LGN) of the thalamus contains a topographic map of the visual field, in which individual neurons represent a small circular region of visual space in the map (Hubel, 1960). Microstimulation (e.g., 40 μA) of LGN neurons can generate phosphenes at the corresponding visual location; and NHPs saccaded to the location of the visual field corresponding to that represented by stimulated neurons (Pezaris and Reid, 2007). Similar effects can be elicited in the motor thalamus, including the ventral anterior/ventral lateral nuclei, where microstimulation can drive contralateral body movements (Vitek et al., 1994). Finally, the ventral posterior lateral sensory nuclei of the thalamus also exhibit somatotopic organization, and microstimulation can drive sensations of body regions corresponding to the receptive fields of stimulated neurons (Swan et al., 2018). Taken together, these data suggest that stimulation via microelectrodes using low currents can exploit the known functional organization of subcortical areas for relatively precise control of sensation and behavior.

## 4 Spatiotemporal-varying stimulation patterns

The standard approach to DBS programming is to increase current sequentially on fully-implanted electrodes and identify the contact (usually one of four or eight) which decreases symptoms without causing side effects. However, there are typically dynamic activation patterns within brain areas that vary according to behavioral demands. Thus, another approach to DBS is to mimic these dynamic activation patterns with stimulation that varies across electrodes and across time.

A key challenge is how to appropriately vary stimulation patterns, in spatial and temporal terms. Spatial variation can be guided by *a priori* knowledge of the internal functional organization of a brain area, which may assist in reducing the parameter space. For example, if a target area contains a functional topographic map, e.g., a somatotopic representation, then optimal stimulation parameters may only need to be sought for those electrodes in the currently relevant functional territory of the map, such as the hand representation for hand movements. Nevertheless, spatially-varied stimulation need not rely on a detailed understanding of the internal functional organization of the target area. Machine learning-based approaches may be sufficient to achieve effective spatiotemporal variation. Suitable spatiotemporal variation may be learned and applied based on both continuous behavioral and neural variables. Such an approach could incorporate prior knowledge of the underlying anatomy, or might simply learn the manifold of responses to a library of stimulation. Thus, the aim would be to identify and then recreate activation patterns for successful cognitive/behavioral performance (see sections 7, 8). It should be noted that the spatial resolution of the stimulation approach (e.g., electrode size) may influence the desired stimulation frequency and temporal variation. For example, the elegant study by Gradinaru et al. (2009) showed that high-frequency (120–130Hz) DBS of the mouse subthalamic nucleus (STN), with a relatively large VTA, reduced Parkinsonian signs; but cell-specific optogenetic stimulation of STN excitatory neurons at the same high, constant frequency did not, suggesting it did not reproduce the same neural dynamics. Rather, optogenetic activation of cortical inputs to the STN reduced Parkinsonian signs, consistent with the efficacy of high-frequency DBS being due to stimulation of the inputs in this case. Here, lowpass filtering of inputs due to synaptic effects (Lavian and Korngreen, 2019; Müller and Robinson, 2018) may contribute to the efficacy of the constant, high-frequency stimulation, whereas direct fine-grained STN stimulation may require more naturalistic stimulation frequencies and spatiotemporal variation. Finally, it is worth noting that current techniques for STN stimulation are not an unqualified success in the treatment of Parkinson's disease; the surgery depends on incredibly precise targeting of the dorsal portion of the nucleus containing the motor cortex afferents (Hamani et al., 2017), and a significant fraction of patients require revision surgery due to lack of benefit (Rolston et al., 2016). Thus, even this flagship example of clinically-successful neuromodulation may benefit from the development of new technology.

## 5 Stimulation effects across brain networks

Because target areas are nodes in an extended brain network, stimulation induces effects at distal network nodes which contribute to behavioral changes and treatment efficacy. Thus, target selection and the spatiotemporal stimulation pattern need tailoring to the (pathological) broader network which we aim to influence, including the intrinsic circuitry. There are high-dimensional cognitive representations in the cerebral cortex distributed over large expanses on the brain's surface, whereas there are more amenable lower-dimensional representations relatively localized in deep brain areas allowing greater generalization (Shine et al., 2019; Mukherjee and Halassa, 2022). A large convergence of anatomical connections from the cortex to deep brain areas leads to the lower dimensionality. These deep brain areas, such as the basal ganglia and thalamus, are key brain hubs connecting distributed groups of cortical neurons, thereby sustaining or flexibly switching cortical activity patterns according to cognitive demands (Saalmann et al., 2012; Schmitt et al., 2017). As each of these brain areas exhibits unique context-dependent neural dynamics, the desired stimulation pattern needs to accordingly mimic these dynamics.

For cortical stimulation, as in neural prostheses, the desired spatiotemporal pattern will differ from that needed for subcortical areas which perform different computations, and affect cortical processes by adjusting synaptic gain and functional connectivity (Guo et al., 2017; Saalmann et al., 2012; Schmitt et al., 2017). Subcortical stimulation may enrich oscillations when they are pathologically impaired (as in the case of coma, see Mofakham et al., 2022; Schmitt et al., 2017), or to counter excessive synchronized/oscillatory activity across a distributed cortical network (Hemptinne et al., 2015). For example, cross-frequency coupling between the phase of low-frequency oscillations and the amplitude of high-frequency oscillations may coordinate distributed cortical processes across a broad network (Canolty and Knight, 2010; Hemptinne et al., 2015). DBS of the STN reduced pathologically elevated beta-band phase-amplitude coupling in the motor cortex (Hemptinne et al., 2015).

## 6 MMBS approach

Two challenges for targeting DBS are the complex-shaped subcortical nuclei and their internal functional organization. Simultaneously stimulating through many small (e.g., <100 μm), closely-spaced (e.g., <200 μm) contacts of multielectrode arrays offers the prospect of tailored manipulation of distinct groups of neurons across brain areas. It is possible to apply different time-varying stimulation parameters to each contact, to exploit the functional organization within the target area (think playing a song on the piano rather than depressing all 88 keys simultaneously). The feasibility of simultaneous microstimulation via multiple electrodes of an array has already been demonstrated in sensory neural prosthesis studies (Mazurek and Schieber, 2021). Here, we first discuss such cortical prostheses, then follow with subcortical studies and approaches using MMBS.

Utah or similar arrays can be used for cortical stimulation. Utah arrays incorporate multiple microelectrodes (shaft length up to a few mm) into an electrode grid (most commonly 10 × 10 shafts) with inter-shaft spacing of 400 μm. Utah arrays have been used for neural prostheses in NHPs (Chen et al., 2020) and humans (Fernández et al., 2021). Stimulation across pairs of electrodes induced phosphenes in humans (Fernández et al., 2021). Simultaneously stimulating up to 16 microelectrodes (the maximum possible with the stimulation system used) could generate more complex perceptions, enabling reliable discrimination of a number of letters of the alphabet. Using multiple Utah arrays (total of 1024 shafts/channels) in NHPs, simultaneous stimulation of up to 15 electrodes across primary visual cortex generated shape information and sequential stimulation generated motion information (Chen et al., 2020). The spatial stimulation pattern exploited the known topographic representation of visual space: specifically, letter information was generated by simultaneously microstimulating those neurons whose visual receptive fields together constituted the letter shape. Together, these data suggest that complex spatially-varying stimulation patterns can mimic natural patterns of neural activation.

The goal stimulation patterns for MMBS will depend on the functional roles of the targeted structure. That is, rather than eliciting a percept, we might attempt to modulate distributed activation patterns across cortical and subcortical areas. For example, higher-order thalamic areas, such as the pulvinar and mediodorsal nuclei, receive little input from the sensory periphery, in contrast to first-order sensory thalamic areas (e.g., LGN). Key functions of the higher-order thalamus appear to be maintenance of cortical activity, such as that supporting action plans and working memory, and regulation of functional connectivity between ensembles of cortical neurons (Guo et al., 2017; Saalmann et al., 2012; Schmitt et al., 2017). This influence is spatially specific, such that subsets of thalamic neurons influence particular ensembles of cortical neurons (Barbas and Pandya, 1989; Schmitt et al., 2017). Thus, MMBS in higher-order thalamus should be able to modulate cortical dynamics on a finer scale than is currently achievable with DBS.

We achieved this level of fine-grained control using MMBS of intralaminar thalamus in NHPs to manipulate consciousness. Major theories of consciousness have proposed that the frontal cortex, parietal cortex and/or thalamus are neural correlates of consciousness (Brown et al., 2019; Llinás et al., 1998; Mashour et al., 2020; Suzuki and Larkum, 2020; Tononi et al., 2016). The intralaminar thalamus has been implicated in the regulation of consciousness, based on lesion (Schiff, 2008) and electrophysiology data (Glenn and Steriade, 1982), and stimulation of intralaminar thalamus using clinical DBS macroelectrodes suggests effects on the level of consciousness (arousal) and/or the level of awareness (richness of experience) (Schiff et al., 2007; Bastos et al., 2021; Tasserie et al., 2022). We targeted the central lateral nucleus (CL) of the intralaminar thalamus, in particular, because it is reciprocally connected with frontal and parietal cortex, and thus CL is well-positioned to influence distributed cortical and subcortical networks. We configured a linear electrode array tailored to the elongated shape of the CL. This allowed us to simultaneously stimulate across 16 contacts (12.5 μm in diameter), with 200 μm between contacts, which spanned the dorsal-ventral extent of CL (3–4 mm in NHPs). In the awake state, a subset of CL neurons fires action potentials at around 50 Hz (Glenn and Steriade, 1982; Redinbaugh et al., 2020), whereas in reduced conscious states, such as general anesthesia and non-REM sleep, the activity of the same cells is reduced. In NHPs anesthetized with propofol or isoflurane, microstimulation across the 16 contacts simultaneously at 50 Hz reinstated wake-like cortical dynamics, including spatially-specific increases in spiking activity in cortical layers and functional connectivity within cortical columns and across parietal and frontal cortex (Afrasiabi et al., 2021; Redinbaugh et al., 2020). These dynamics corresponded to the rousing of NHPs from anesthesia. The effect was specific to CL stimulation, as moving the stimulating array 1.5 mm from CL did not have the same effect. Neither did stimulation at 2, 10, or 200 Hz. This suggests that mimicking the wake-like activity of CL neurons with 50 Hz MMBS overrode the neural and behavioral effects of anesthesia.

We can also reduce the level of consciousness with this approach. We performed the same experiments in awake NHPs (Redinbaugh et al., 2022). Microstimulating via the 16 CL contacts at 10 or 200 Hz reduced consciousness, inducing absence-like events with vacant staring, behavioral arrest, and reduced responsiveness. Stimulation at 50 Hz did not have the same effect. This frequency selectivity may reflect the fact that a subset of CL neurons naturally decrease their firing rate from about 50 Hz in the wake state nearer to 10 Hz in reduced conscious states (Redinbaugh et al., 2020), consistent with predictions from the mesocircuit model of Nicholas Schiff (2010). In comparison, 200 Hz stimulation might perturb CL activity in wakefulness, by inducing bursting-like activity followed by inhibition (Birdno et al., 2014; Patra, 2001), perhaps similar to that seen in sleep/anesthesia. These findings suggest that mimicking the activity of CL neurons in reduced conscious states with 10 Hz or 200 Hz MMBS overrode wakeful cortical dynamics and behavior. This bidirectional control of the level of consciousness in NHPs by mimicking CL activity in different conscious states is consistent with optogenetic effects in mice, seen when stimulating CL neurons at similar frequencies (10, 40, and 100 Hz) (Liu et al., 2015).

To achieve this kind of spatial specificity, there are a number of existing DBS electrodes with large numbers of small-diameter contacts, such as the μDBS electrode (Anderson et al., 2019). Most are not FDA-approved, however. The ideal MMBS electrode should allow independent control over all or a subset of contacts, and be capable of simultaneous stimulation (and recording). Proposed electrodes vary in contact size and geometry (Rossi et al., 2016). Linear microelectrode arrays have been used in animal studies to manipulate thalamic targets; the linear arrays used in the above CL studies are configurable with up to 32-contact shafts, 12.5–50 μm contact diameters and inter-contact spacing of 75–250 μm (from MicroProbes for Life Science™: Gaithersburg, MD). These electrodes are suitable for anisotropic, elongated structures like CL. Multiple shafts or microwires may be better suited for isotropic brain structures.

Microwires have been used in humans for both recording and stimulation (Fried et al., 1997; Swan et al., 2018). In a study of human somatosensory thalamus, microstimulation via individual wires of a 16-channel microwire array (Ad-Tech™: Oak Creek, WI) elicited localized sensations (1–3 cm^2^) on the body, whereas stimulation via macroelectrodes (Medtronic™: Dublin, Ireland) elicited considerably more extensive sensations (e.g., entire forearm; compare Fig. 2 and 3 to Fig. 5 in (Swan et al., 2018)). Research subjects described the microwire-elicited sensations as more “natural” than stimulation via macroelectrodes. However, one has to account for variability in the dispersion of microwires and the possible addition of more microwire arrays to cover the entire target area.

Silicon-based designs (HajjHassan et al., 2008) offer higher contact counts, which could meet this need. One electrode design uses silicon chips fitted together to form an extruded plus-shaped electrode (Willsie and Dorval, 2015) with 1760 contacts (four contacts on each plus-shaped cross-section). Each individual contact is 100 × 100 μm, with adjacent contacts spaced 115 μm. The contact size was suggested to correspond to the scale of small fiber tracts. A variant of this electrode design (Anderson et al., 2019) incorporates 864 contacts, with 150 × 150 μm individual contacts and adjacent contacts spaced 15 μm. In both cases, individual contacts are independently controllable, and current can be applied via multiple contacts simultaneously. The overall diameter of the electrodes is about 1.27 mm, similar to FDA-approved DBS electrodes. Simulations of stimulation effects on small and large axons suggest that the small contact size and inter-contact spacing more readily enable activation of small axons (relative to macroelectrodes) (Anderson et al., 2019; Steinmetz et al., 2018). However, as the number of electrodes increases, the parameter space increases exponentially, leading to a need for a computational technique to optimize programming.

## 7 Leveraging machine learning

Machine learning is an attractive strategy to guide DBS programming, because of the ability to efficiently explore spaces of unknowns. Moreover, tuning stimulation to the brain's state is straightforward to conceptualize as an optimization problem. Machine learning has been successfully applied to decoding cortical activity from electrode arrays for neural prostheses (Anumanchipalli et al., 2019; Willett et al., 2021). Machine learning has also started to be applied for optimizing stimulation parameters using clinical DBS electrodes, i.e., via one or two relatively large contacts (Boutet et al., 2021; Gao et al., 2020; He et al., 2021; Houston et al., 2019; Krylov et al., 2020; Yang et al., 2021). However, simultaneous multi-microelectrode stimulation is relatively new, and thus machine learning has not yet been applied to optimizing stimulation patterns across multi-electrode arrays, let alone in a closed-loop system to improve behavioral outcomes. To advance in this direction, we first discuss existing closed-loop approaches to stimulation. We then describe the computational framework for closed-loop stimulation across multi-microelectrode arrays.

A number of closed-loop approaches to DBS have been reported, although only the Neuropace Responsive Neurostimulation (RNS) system is available commercially (the PERCEPT/SenSight DBS system is currently available as open-loop in the US). The RNS stimulates cortical targets in response to detected seizures. The exact algorithms for seizure detection are proprietary but are believed to include relatively simple approaches based on spike detection, band-limited power, and line length (Sun et al., 2008). Using closed-loop stimulation, RNS decreases seizures by ∼50% in most patients (Bergey et al., 2015). RNS has also been used off-label for depression (Scangos et al., 2021) and is planned for use in binge eating (Wu et al., 2022, 2018).

However, despite progress in closed-loop stimulation for Parkinson's disease (Rosin et al., 2011) and essential tremor (Opri et al., 2020), no other closed-loop brain stimulator is available commercially. Moreover, the diseases treated thus far have relatively straightforward electrophysiological biomarkers and respond to relatively coarse stimulation, using large contact electrodes in well-described targets. In no case was there a treatment involving a neuroanatomical location that responded *only* to closed-loop stimulation; open-loop stimulation has been reported for all of the above diseases. The next generation of DBS for complex problems (such as cognitive impairment, or unconsciousness) will depend on high-resolution stimulation of specific populations of neurons as well as careful tuning to observed activity, which is possible only with closed-loop stimulation.

There are two possible approaches to closed-loop stimulation: deciding whether to stimulate based on a single feature such as amplitude (like RNS, as described above), or stimulating based on a more sophisticated analysis of the brain's latent state. The estimation of the cognitive state is often carried out within the state-space modeling framework, under the assumption that the cognitive state is a low-dimensional dynamic latent process that can be estimated (Abbaspourazad et al., 2021; Cueva et al., 2020; Sani et al., 2018).

**Fig. 1:**
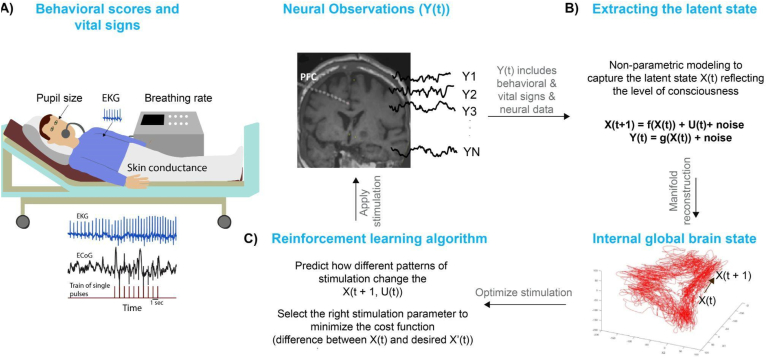
**Schematic of closed-loop stimulation for recovery of consciousness. A)** high-dimensional observations Y(t) includes deep neural recordings from distributed contacts and vital signs (heart rate, pupil size). **B)** Non-parametric state-space modeling enables us to estimate the underlying latent state of these observations at baseline and with different sets of stimulation parameters. **C)** Reinforcement learning will determine which set of stimulation parameters will minimize our cost function, that is the distance of the predicted X(t, u(t)) from the healthy, desired X′(t).

The task of estimating cognitive states is quite challenging because they cannot be observed directly. There are two steps needed to estimate cognitive states: finding the underlying manifold of the cognitive state and decoding how brain signals relate to this manifold. Yousefi and colleagues used state-space modeling to form low-dimensional manifolds of internal cognitive states from behavioral features (Yousefi et al., 2019). The next step is building an “encoder/decoder,” i.e., mapping the multiple features of the neural and behavioral signals to a cognitive state/mapping the behavioral state to features of neural and behavioral signals. The most critical feature of the encoder/decoder is specification of a model (mapping) for the cognitive state. This model is often parametric and involves a number of parameters and various explanatory variables. Often, it is necessary to build several candidate models. The building of the encoder/decoder then involves finding the optimal parameters of the model followed by selecting the best model (Yousefi et al., 2019, 2018). Finding the optimal parameters requires an optimization procedure of the encoder/decoder, and model selection amounts to identifying the best model from the explored models using a predefined criterion. The used criterion should encourage the selection of a parsimonious model. The problem of estimating the cognitive state process can be cast within the Bayesian framework, which then becomes one of estimating the posterior distribution of the cognitive state, given the observed neural and behavioral features, and using the adopted form of the encoder/decoder. It is important to note that the cognitive state is a time-varying process, which implies that the posterior distribution of the hidden state has to be estimated sequentially in real-time. This approach is schematized in Fig. 1.

A simple description of evolution of cognitive states with respect to electrical stimulation using state space models requires two equations:

(Eq. 1) X(t+1) = f(X(t)) + U(t) + noise

(Eq. 2) Y(t) = g(X(t)) + noise

These two equations describe the time evolution of the internal brain states (Eq. (1)) and the observed signals (Eq. (2)). Where the observations (Y(t) in Eq. (2)) are high dimensional signals that could be neural recordings (LFP, ECoG, IEEG) or behavioral features (task-specific features, vital signs, etc.) or the combination of the two (Fig. 1A). From these observations Y(t) one can estimate the latent brain state X(t) **(**Eq. (2), Fig. 1B**)***.* Here, external stimulation can be considered an arbitrary perturbation U(t). Here, X(t) represents a low-dimensional latent state of the global brain activity (Fig. 1B), “f” is a general nonlinear function that describes how this latent state evolves over time. The symbol “g” quantifies the relationship between the latent state and the observations.

The ultimate goal of closed-loop stimulation is to apply intelligent stimulation U(t) to drive the internal brain state X(t) toward the desired healthy space X′(t) on the extracted low-dimensional manifold. To do that we need to learn “f” and “g” from the observed data. One way of estimating these functions is by treating them as random objects whose priors are Gaussian processes (Wang et al., 2008). One can show that the underlying dynamics of the cognitive state and the mappings of the state to the observed signals can be learned by several suitable Bayesian, non-parametric approaches including Gaussian processes (GPs) and deep Gaussian processes (DGPs) combined with particle filtering (Liu et al., 2022). These non-parametric approaches have a clear advantage over previously developed parametric attempts that have assumed conditional linear models in Eq. (1) and Eq. (2)**.** Yang and colleagues reported a similar approach to predicting stimulation responses using linear models, where the neural state Y(t) was conceptualized as a combination of ongoing activity, stimulation-evoked activity, and noise (Yang et al., 2021). However, the reliance on linear models is a drawback of this approach.

GPs are a fully Bayesian technique for learning an underlying function governing a given process (i.e., LFP over time) with the assumption that the indexed random variables have an underlying joint Gaussian or normal distribution (Kuss and Rasmussen, 2003; Younes and Panov, 2019). Deep Gaussian processes (DGPs) are a deep-learning analog of GPs, where multiple GPs are layered and sequentially learn different features to optimize the learning of the function (Ghavamzadeh et al., 2015). The non-parametric nature of the GPs makes them very powerful in determining the unknown functions “f” and “g”. Further, we can even allow these functions to change as a function of time, to reflect brain dynamics. Lastly, to increase the capacity of the modeling, one can utilize a large ensemble of deep GPs, where each deep GP works with its idiosyncratic kernels and processes the recorded electrophysiological signals independently. Then the results of each deep GP are fused to obtain the final result (Liu et al., 2022).

Particle filtering is a methodology that can complement the approaches based on GPs and DGPs. Namely, a particle filter generates many versions of the latent process (by sampling from a proposal distribution) and to each version, it assigns a weight that reflects the likelihood of the version being correct. The ensemble of deep GPs will operate in a similar way. In other words, there will be many GPs exploring candidate functions “f” and “g”, and they will all be scored based on how they represent the observed LFP signals as in particle filtering.

## 8 Machine learning-guided MBBS for cognitive enhancement

In this section, we discuss machine learning-guided MMBS applications to disorders of consciousness and disorders of cognitive control. In particular, stimulating CL to enhance consciousness and stimulating mediodorsal thalamus (MD) to improve cognitive control may require somewhat different approaches. In particular, effective regulation of cognitive control may require spatiotemporal variation of MD microstimulation on the scale of seconds or faster. More coarse stimulation of CL may be sufficient to increase the level of consciousness, given diurnal fluctuation and the physiological properties and organization of neurons in CL.

For disorders of consciousness, such as coma or minimally conscious state, treatment will require different goal conditions in the thalamus and cortex. In the thalamus, restoration of elevated, wake-like (e.g., 50 Hz) CL firing is likely sufficient; in the cortex, it will be necessary to restore long-range cortico-cortical communication as well as communication between superficial and deep cortical layers (Redinbaugh et al., 2020). It was sufficient in our previous study to use the same stimulation parameters across all contacts. More physiological stimulation patterns are possible, such as Poisson-distributed stimulation to better reflect natural spiking dynamics. Other, customized approaches are possible too; as described in section 7, non-parametric approaches such as GPs can be used to predict cortical responses to CL stimulation. Based on these predictions, reinforcement learning (RL) can help select the stimulation pattern that results in a cortical response which minimizes the cost function (Fig. 1C). For an RL approach, electrophysiological and behavioral biomarkers can be used to create the cost function. A more complex approach is needed to restore cognitive control.

Cognitive control is the ability to flexibly adapt behavior according to goals and context. Numerous conditions including TBI, autism, and schizophrenia have deficits in cognitive control. The prefrontal cortex (PFC) is vital for cognitive control, and neuroimaging evidence suggests that pathological processes affecting the PFC in TBI predominantly contribute to the cognitive control deficits. Altered PFC activations following TBI (Olsen et al., 2015; Scheibel, 2017) may reflect compensatory mechanisms toward effective cognitive control. The PFC is strongly interconnected with subcortical areas, e.g., MD, which also play vital roles in cognitive control. MD helps maintain ongoing activity in (mouse) PFC and contributes to flexible activations of PFC neuronal ensembles, important for attentional processing and task-switching. Recent evidence suggests that the MD influences the recovery of TBI patients (Mofakham et al., 2022, 2021).

MD stimulation is thus a reasonable MMBS target for restoration of cognitive control. MD has specific topographical connectivity with PFC (Phillips et al., 2019). This topographic connectivity can be exploited by implanting recording electrodes across PFC and recording/microstimulating electrodes across MD. These recordings would allow identification of atypical PFC sites and their connected MD sites. Current could then be applied through the corresponding subset of MD microelectrodes, to normalize activity within PFC sites. A number of electrophysiological biomarkers might be used to guide MMBS of MD to induce PFC dynamics supporting improved cognitive control. Increased spiking of PFC neurons and bursts of gamma (30–80Hz) activity support working memory (Miller et al., 2018), a core component of cognitive control (Gonzalez-Burgos et al., 2015). Work in mice suggests that after initial activation of PFC neurons during a cognitive task, sustained activity of ensembles of PFC neurons across a delay relies on MD (Schmitt et al., 2017). Further, synchrony in the beta frequency range (13–30Hz) between MD and PFC increases during working memory (Parnaudeau et al., 2013). Thus, suitable biomarkers might include gamma activity and beta coherence as well as sustained firing in PFC, which could be estimated from spiking or high gamma responses.

Because lesions are rarely circumscribed to a particular brain area, it may be beneficial to manipulate multiple areas to restore affected functions. For example, in cases presenting with impaired consciousness and cognitive control, MMBS of both CL and MD may have complementary effects. Intelligent stimulation, guided by the Bayesian non-parametric approaches in section 7, provides a means to optimize MMBS across areas to achieve desired brain states.

## 9 Conclusion

We have espoused the view that the next generation of MMBS technology will use a closed-loop, machine learning-based approach. These technologies will both improve on treatments for diseases currently treated by neuromodulation, and enable the treatment of diseases that are not currently amenable to neuromodulation. Current devices are purpose-built and able to stimulate one or a few contacts; future devices with many smaller contacts will stimulate multiple areas simultaneously to drive pathological brain states to healthy states. Of course, the spatial and temporal space for neuromodulation is large, almost intractable; we propose that machine learning approaches will be critical to these future developments. To fully operationalize the technology, a number of advances in technology (both hardware and software), and in understanding the brain's response to stimulation will be critical. To develop the proposed technology, the next steps are NHP studies, which will be critical for both hardware and software development. Early translation into human studies should then be pursued, given the overwhelming need for new treatments for neurological and psychiatric diseases.

## CRediT authorship contribution statement

**Yuri B. Saalmann:** Conceptualization, Writing – original draft, Writing – review & editing, Supervision, Funding acquisition. **Sima Mofakham:** Conceptualization, Methodology, Writing – original draft, Writing – review & editing, Visualization, Funding acquisition. **Charles B. Mikell:** Conceptualization, Writing – original draft, Writing – review & editing, Funding acquisition. **Petar M. Djuric:** Conceptualization, Methodology, Writing – original draft, Writing – review & editing, Funding acquisition.

## Declaration of competing interest

The authors declare that they have no known competing financial interests or personal relationships that could have appeared to influence the work reported in this paper.

## Data Availability

No data was used for the research described in the article.
