# Supplementary material for: Microscale multicircuit brain stimulation: Achieving real-time brain state control for novel applications
Source: Curr Res Neurobiol. 2022 Dec 29;4:100071. doi: 10.1016/j.crneur.2022.100071 (PMC9816916; doi:10.1016/j.crneur.2022.100071)
Supplement: Multimedia component 1 [file mmc1.pdf]

Peer Review Overview

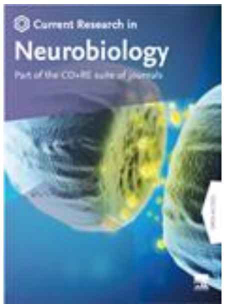

Manuscript Title: Microscale multicircuit brain stimulation: achieving real-time brain state control for novel applications

Received	Aug 30, 2022
1st Decision	Oct 17, 2022
1st Revision Submitted	Nov 30, 2022
Accepted	Dec 19, 2022

1st Decision letter

Reference: CRNEUR-D-22-00048
Title: Microscale multicircuit brain stimulation: achieving real-time brain state control for novel applications
Journal: Current Research in Neurobiology

Dear Prof Saalmann,

Thank you for submitting your manuscript to Current Research in Neurobiology.

I have completed my evaluation of your manuscript. The reviewers recommend reconsideration of your manuscript following revision. I invite you to resubmit your manuscript after addressing the comments below. Please resubmit your revised manuscript by Dec 01, 2022.

When revising your manuscript, please consider all issues mentioned in the reviewers' comments carefully: please outline every change made in response to their comments and provide suitable rebuttals for any comments not addressed. Please note that your revised submission may need to be re-reviewed.

Current Research in Neurobiology values your contribution and I look forward to receiving your revised manuscript.

Kind regards,

Anna S Mitchell, Ph.D.
Editor in Chief
Current Research in Neurobiology

Comments from Editors and Reviewers:

Reviewer #1:

Anderson et al 2019 is cited as both an 'a' and a 'b' version. Presuming that the authors intended to cite two different papers, they should update the References accordingly.

Reviewer #2:

Saalmann et al. introduce a new concept for electrical modulation of the brain, named microscale multicircuit brain stimulation (MMBS). They suggest MMBS as an alternative to the current neuromodulation approach that became popular in the clinical neurosciences, namely deep brain stimulation (DBS). They examine the concrete example of consciousness restoration with thalamic stimulation to establish their new cocept, implementing tiny electrodes, closed-loop approaches with massive recording of ensemble of neurons and a mathematical framework that extracts the latent space and build internal global brain states.
This is a very nice paper with highly ambitious purposes that may end up changing the current paradigm of clinical brain stimulation approaches.
However I must say that there is a crucial need for further substantial explanations and discussions.

1-The authors constantly refer to DBS pinpointing its coarse stimulation as opposed to fine-grained MMBS. It should be noted that DBS is currently the only neuromodulation approach that provides striking shift in behavior and quality of life of patients with Parkinson's disease, essential tremor and dystonia. Importantly, a growing literature shows that the effect and mechanisms of DBS act through cortical modulation (for example Gradinaru et al, Science, 2009; de Hemptinne et al., Nature Neurosci, 2015). This has specifically been demonstrated recently for DBS effects on consciousness restoration (Tasserie et al., Sci Adv, 2022). The authors must dedicate a section to discuss the cortical mechanisms of DBS and the consequences for the MMBS concept.
It has tremendous consequences on the network that MMBS should target. For example, Gradinaru paper used fine-grained optogenetic with high cell subtype specificity to demonstrate that stimulating the subthalamic neurons don't have an effect on behavior, whereas the modulation of the cortico-subcortical pathway does! With its VTA, DBS can modulate the targeted nucleus AND the afferent/efferent pathways, thus achieving massive cortical modulation of remote areas, and leading to Parkinson's correction.
The risk of microstimulating the same area (STN) is to lose the effect ! In that case, MMBS should target the cortical network instead. VTA can have a positive consequence.
This is a major potential limitation of the approach and I deeply recommend that the authors add a whole section to discuss thoroughly the cortico-subcortical interactions, ideally with a n additional figure.
Moreover, authors present MMBS as the fine-grained approach to brain stimulation. They should also discuss the potential of optogenetics to achieve even higher specificity (cell type) then MMBS.

2-Consciousness paragraph (lines 180-200):
The authors should introduce the concept of consciousness with its two different aspects: arousal/vigilance AND awareness/conscious access. And with a current overview on theories of consciousness with their influence on the rationale for brain stimulation (IIT, GNW, TCL, and more recent work by Larkum group). In fact these are two different levels of consciousness, driven by completely different brain networks, and that need different investigation approaches. The aim of DBS is

to ameliorate the two aspects and not only arousal/wakefulness, to be clinically relevant. In a recent paper, Tasserie et al, (Sci Adv, 2022), could demonstrate the restoration of the two different levels of consciousness with central thalamic DBS. Here again Tasseire et al. show that the restoration of consciousness went through key cortical modulation. Redinbaugh et al also did demonstrate the importance of cortical modulation during thalamic stimulation.
The authors should significantly improve this section dedicated to consciousness studies.

3-Clinical applications:
The authors shoud discuss the potential increase in invasiveness with MMBS. In fact, MMBS may puch the neurosurgeon to operate on multiple parts of the brain, thus increasing the neurosurgical risks. Another aspect is the stimulation programming. It is very unclear how to practically implement the customized electrical settings. The authors pinpoint the importance of closed-loop stimulation as an innovative aspect of MMBS. However they forgot to mention that closed-loop/adaptive stimulation already exist with the recent PERCEPT/SenSight DBS system of Medtronic, with a recent case report of its application in Parkinson's (Nakajima et al., Front Hum Neurosci, 2021).

1st Author Response Letter

Response to comments from Editors and Reviewers:

We would like to thank the reviewers for their thoughtful comments and useful feedback towards improving our manuscript. The reviews of the manuscript were positive and made suggestions for improvement. Reviewer #1 asked us to correct referencing. Reviewer #2 asked that we further discuss DBS mechanisms, including cortical mechanisms, cortico-subcortical interactions and VTA effects; elaborate on consciousness theory and its relation to DBS; and address comments on the clinical application of MMBS. We have revised the manuscript accordingly, including adding a new section to the manuscript as requested, and responded to reviewers point-by-point below.

Comments from Reviewer 1

Anderson et al 2019 is cited as both an 'a' and a 'b' version. Presuming that the authors intended to cite two different papers, they should update the References accordingly.

We thank the reviewer for pointing this out. We intended to cite the same paper by Anderson et al. (2019). It erroneously appeared twice in the reference list. This has now been corrected.

Comments from Reviewer 2

Saalmann et al. introduce a new concept for electrical modulation of the brain, named microscale multicircuit brain stimulation (MMBS). They suggest MMBS as an alternative to the current neuromodulation approach that became popular in the clinical neurosciences, namely deep brain stimulation (DBS). They examine the concrete example of consciousness restoration with thalamic stimulation to establish their new cocept, implementing tiny electrodes, closed-loop approaches with massive recording of ensemble of neurons and a mathematical framework that extracts the latent space

and build internal global brain states. This is a very nice paper with highly ambitious purposes that may end up changing the current paradigm of clinical brain stimulation approaches. However I must say that there is a crucial need for further substantial explanations and discussions.

1a-The authors constantly refer to DBS pinpointing its coarse stimulation as opposed to fine-grained MMBS. It should be noted that DBS is currently the only neuromodulation approach that provides striking shift in behavior and quality of life of patients with Parkinson's disease, essential tremor and dystonia.

We agree with the reviewer that this should be noted in the text. Accordingly, we have now added the following text to the manuscript in section 1. Introduction (page 3): "It is important to note that DBS robustly improves quality of life of patients with Parkinson's disease, essential tremor and dystonia."

1b-Importantly, a growing literature shows that the effect and mechanisms of DBS act through cortical modulation (for example Gradinaru et al, Science, 2009; de Hemptinne et al., Nature Neurosci, 2015). This has specifically been demonstrated recently for DBS effects on consciousness restoration (Tasserie et al., Sci Adv, 2022). The authors must dedicate a section to discuss the cortical mechanisms of DBS and the consequences for the MMBS concept.

We agree with the reviewer that thalamic stimulation is effective because of resulting cortical effects and effects on broader brain networks. As the reviewer suggested, we have added a new section, "5. Stimulation effects across brain networks", which discusses the cortical effects of DBS. The text in this section is: "Because target areas are nodes in an extended brain network, stimulation induces effects at distal network nodes which contribute to behavioral changes and treatment efficacy. Thus, target selection and the spatiotemporal stimulation pattern need tailoring to the (pathological) broader network which we aim to influence, including the intrinsic circuitry. There are high-dimensional cognitive representations in the cerebral cortex distributed over large expanses on the brain's surface, whereas there are more amenable lower-dimensional representations relatively localized in deep brain areas allowing greater generalization (Shine et al., 2019; Mukherjee and Halassa, 2022). A large convergence of anatomical connections from the cortex to deep brain areas leads to the lower dimensionality. These deep brain areas, such as the basal ganglia and thalamus, are key brain hubs connecting distributed groups of cortical neurons, thereby sustaining or flexibly switching cortical activity patterns according to cognitive demands (Saalmann et al., 2012; Schmitt et al., 2017). As each of these brain areas exhibits unique context-dependent neural dynamics, the desired stimulation pattern needs to accordingly mimic these dynamics.

For cortical stimulation, as in neural prostheses, the desired spatiotemporal pattern will differ from that needed for subcortical areas which perform different computations, and affect cortical processes by adjusting synaptic gain and functional connectivity (Guo et al., 2017; Saalmann et al., 2012; Schmitt et al., 2017). Subcortical stimulation may enrich oscillations when they are pathologically impaired (as in the case of coma, see (Mofakham et al., 2022; Schmitt et al., 2017)), or to counter excessive synchronized/oscillatory activity across a distributed cortical network (Hemptinne et al., 2015). For example, cross-frequency coupling between the phase of low-frequency oscillations and the amplitude of high-frequency oscillations may coordinate distributed cortical processes across a broad

network (Canolty and Knight, 2010; Hemptinne et al., 2015). DBS of the STN reduced pathologically elevated beta-band phase-amplitude coupling in the motor cortex (Hemptinne et al., 2015)."

1c-It has tremendous consequences on the network that MMBS should target. For example, Gradinaru paper used fine-grained optogenetic with high cell subtype specificity to demonstrate that stimulating the subthalamic neurons don't have an effect on behavior, whereas the modulation of the cortico-subcortical pathway does! With its VTA, DBS can modulate the targeted nucleus AND the afferent/efferent pathways, thus achieving massive cortical modulation of remote areas, and leading to Parkinson's correction.
The risk of microstimulating the same area (STN) is to lose the effect ! In that case, MMBS should target the cortical network instead. VTA can have a positive consequence.
This is a major potential limitation of the approach and I deeply recommend that the authors add a whole section to discuss thoroughly the cortico-subcortical interactions, ideally with a n additional figure.

We agree that the effects of DBS via macroelectrodes and more fine-grained stimulation can have different outcomes, especially if the stimulation parameters are not tailored to each technique. The excellent Gradinaru et al. paper showed optogenetic stimulation of STN excitatory neurons – using the same high-frequency (120-130Hz) stimulation as DBS – did not recreate the same neural dynamics nor behavioral outcome as DBS. One possible reason for this is that the fine-grained stimulation needs to more closely mimic the typical neural dynamics of the target area. This is the motivation for machine-learning-guided MMBS. What we are arguing is that a relatively homogenous, large VTA is not mimicking healthy physiology. If a relatively large VTA reduces symptoms, e.g., in Parkinson's Disease, then this is clearly a positive clinical outcome. Nonetheless, we still lack effective interventions in a number of neuropsychiatric disorders. In thinking about other approaches to DBS, we agree that classical microstimulation might lead to a very small VTA and may not have a clinical effect. What we are proposing is an approach in which there are many microstimulated sites, each with its own stimulation dynamics, which can occupy an extended region of the brain. This approach would aim to mimic healthy physiological activity better than a homogenous large VTA.

We appreciate the reviewer's suggestion that the manuscript would be improved by further discussion of these issues. Accordingly, we have added text to clarify how the VTA by DBS influences outcomes, including description of the Gradinaru et al. data. In addition to the new section 5. "Stimulation effects across brain networks" (see response to reviewer's comment 1b), we have now added the following text to the manuscript in section 4. Spatiotemporal-varying stimulation patterns (page 6): "It should be noted that the spatial resolution of the stimulation approach (e.g., electrode size) may influence the desired stimulation frequency and temporal variation. For example, the elegant study by Gradinaru et al. (2009) showed that high-frequency (120-130Hz) DBS of the mouse subthalamic nucleus (STN), with a relatively large VTA, reduced Parkinsonian signs; but cell-specific optogenetic stimulation of STN excitatory neurons at the same high, constant frequency did not, suggesting it did not reproduce the same neural dynamics. Rather optogenetic activation of cortical inputs to the STN reduced Parkinsonian signs, consistent with the efficacy of high-frequency DBS being due to stimulation of the inputs in this case. Here, lowpass filtering of inputs due to synaptic effects (Lavian and Korngreen, 2019; Müller and Robinson, 2018) may contribute to the efficacy of the constant, high-frequency stimulation, whereas direct fine-grained STN stimulation may require more naturalistic stimulation

frequencies and spatiotemporal variation. Finally, it is worth noting that current techniques for STN stimulation are not an unqualified success in the treatment of Parkinson's disease; the surgery depends on incredibly precise targeting of the dorsal portion of the nucleus containing the motor cortex afferents (Hamani et al., 2017), and a significant fraction of patients require revision surgery due to lack of benefit (Rolston et al., 2016). Thus, even this flagship example of clinically-successful neuromodulation may benefit from the development of new technology."

1d-Moreover, authors present MMBS as the fine-grained approach to brain stimulation. They should also discuss the potential of optogenetics to achieve even higher specificity (cell type) then MMBS.

We thank the reviewer for the suggestion of comparing MMBS with optogenetics. Optogenetics offers targeting of particular cell types, but all targeted cells are stimulated at the same frequency through the optical fiber. To independently manipulate different cell groups within a brain area, multiple optical fibers would have to be inserted into the area, each fiber with its own size/resolution limits, and each requiring its own light source. Further, MMBS can be more readily translated to the clinic compared with the viral techniques involved in optogenetics. We have now added the following text to the manuscript in section 3. What spatial resolution is possible? (page 4): "The currently available methods for manipulating neural activity up to high frequencies are electrical stimulation and optogenetics. While optogenetics offers cell-type specificity, there are drawbacks. First, optogenetics faces limits in spatiotemporally varying control, i.e., every new group of neurons to be independently controlled would need a new optical fiber, with its own size limits, and light source. Second, there is the potential for immunogenicity (Mendoza et al., 2017). Third, advances in rodents have not readily translated to primates (Servick, 2020). Finally, optogenetics currently uses repetitive, constant-frequency stimulation patterns, which do not resemble nor induce the dynamic activity patterns in the brain necessary for cognition. In comparison, electrical stimulation is currently applied as either DBS using one or few large electrode contacts (around 1mm diameter) with one large VTA (>10mm^3; Butson et al., 2007; Dembek et al., 2017), orders of magnitude poorer than the necessary spatial resolution to independently control individual groups of neurons to enhance cognition; or highly localized microstimulation via one or few microelectrodes, which targets one group of neurons (Nichols and Newsome, 2002; Mazurek and Schieber, 2021) but not other groups distributed over large expanses of the brain which contribute to distributed cognitive computations. This latter issue, however, can be overcome by using a high number of microelectrodes spanning one or more brain areas, i.e., the basis of the MMBS approach, and our focus hereon."

2-Consciousness paragraph (lines 180-200):
The authors should introduce the concept of consciousness with its two different aspects: arousal/vigilance AND awareness/conscious access. And with a current overview on theories of consciousness with their influence on the rationale for brain stimulation (IIT, GNW, TCL, and more recent work by Larkum group). In fact these are two different levels of consciousness, driven by completely different brain networks, and that need different investigation approaches. The aim of DBS is to ameliorate the two aspects and not only arousal/wakefulness, to be clinically relevant. In a recent paper, Tasserie et al, (Sci Adv, 2022), could demonstrate the restoration of the two different levels of

consciousness with central thalamic DBS. Here again Tasseire et al. show that the restoration of consciousness went through key cortical modulation. Redinbaugh et al also did demonstrate the importance of cortical modulation during thalamic stimulation.
The authors should significantly improve this section dedicated to consciousness studies.

We appreciate the reviewer's suggestion. Accordingly, in the manuscript we have introduced (i) the concepts of level of consciousness (arousal) and level of awareness (richness of experience) in relation to thalamic DBS effects; (ii) fronto-parietal and thalamic neural correlates of consciousness proposed by major theories of consciousness; and (iii) further clarified the rationale for targeting the intralaminar thalamus for DBS in disorders of consciousness. This includes citing the excellent paper from Tasserie et al. (which was published after our original submission) as well as the other relevant DBS and theories of consciousness papers. To be clear, we agree completely with the reviewer that thalamic stimulation is effective because of resulting cortical effects as described above in response to the reviewer's comment 1b and in the manuscript in relation to thalamic stimulation effects on cortex in the modulation of consciousness: "…reinstated wake-like cortical dynamics, including spatially-specific increases in spiking activity in cortical layers and functional connectivity within cortical columns and across parietal and frontal cortex". We have now added the following text to the manuscript (p. 9): "Major theories of consciousness have proposed that the frontal cortex, parietal cortex and/or thalamus are neural correlates of consciousness (Brown et al., 2019; Llinás et al., 1998; Mashour et al., 2020; Suzuki and Larkum, 2020; Tononi et al., 2016). The intralaminar thalamus has been implicated in the regulation of consciousness, based on lesion (Schiff, 2008) and electrophysiology data (Glenn and Steriade, 1982), and stimulation of intralaminar thalamus using clinical DBS macroelectrodes suggests effects on the level of consciousness (arousal) and/or the level of awareness (richness of experience) (Schiff et al., 2007; Bastos et al., 2021; Tasserie et al., 2022). We targeted the central lateral nucleus (CL) of the intralaminar thalamus, in particular, because it is reciprocally connected with frontal and parietal cortex, and thus CL is well-positioned to influence distributed cortical and subcortical networks."

3-Clinical applications:
The authors shoud discuss the potential increase in invasiveness with MMBS. In fact, MMBS may puch the neurosurgeon to operate on multiple parts of the brain, thus increasing the neurosurgical risks.
Another aspect is the stimulation programming. It is very unclear how to practically implement the customized electrical settings. The authors pinpoint the importance of closed-loop stimulation as an innovative aspect of MMBS. However they forgot to mention that closed-loop/adaptive stimulation already exist with the recent PERCEPT/SenSight DBS system of Medtronic, with a recent case report of its application in Parkinson's (Nakajima et al., Front Hum Neurosci, 2021).

The reviewer raises important considerations. We do not expect MMBS to increase invasiveness. Clinical DBS can involve bilateral stimulation, with the diameter of each electrode around 1.3mm (e.g., Schiff et al., 2007, Nature). MMBS can also involve bilateral stimulation, with current linear electrode arrays suitable for MMBS smaller or the same size (e.g., microDBS probe from Anderson et al., 2019) as current clinical DBS electrodes. The relatively large number of contacts on the MMBS electrode may span more than one deep brain area, but the contacts would all be part of the same electrode. Thus,

this would not increase the neurosurgical risks. Discussion of the size of the suggested MMBS electrodes is in section “6. MMBS approach”.

The closed-loop PERCEPT/SenSight DBS system reported in Nakajima et al. (2021) adapts the stimulation current depending on the amplitude of beta-frequency neural oscillation. While this approach (detection of large beta oscillations and stimulation via one/few macroelectrodes) has advantages over conventional DBS, we are proposing a closed-loop approach based on non-parametric state-space modeling and stimulation through many microelectrodes. Unlike the approach in Nakajima et al. which decides whether to stimulate based on a single feature (beta oscillations), our approach aims to predictively estimate the brain’s latent state (based on real-time behavioral and neural indicators), and use reinforcement learning to determine stimulation parameters across our MMBS electrode array (to minimize the distance of the predicted state from the healthy, desired state). This is a very different computational and hardware strategy compared with current clinical DBS approaches (including Nakajima et al.). As discussed in the manuscript, Yang et al. (2021) have used a similar computational approach, but based on linear models (and macroelectrode stimulation), offering some support for the feasibility of our machine-learning-guided MMBS approach.

Although the PERCEPT/SenSight DBS system of Medtronic is available in the US for open-loop clinical use (and is used clinically by author CM), the closed-loop function is not yet available (to the best-of-our-knowledge, the company plans to roll out this functionality in the future). We have added this comment to the manuscript on page 13.

Regarding stimulation programming, the ultimate goal is to apply intelligent stimulation to drive the brain’s latent state to the desired state. In the approach we propose, this requires learning the underlying dynamics of the cognitive state and the mapping of the state to the observed signals. One strategy to do this is to record behavioral and neural activity at baseline, and with different sets of stimulation parameters, after surgery while the patient is in the hospital. The initial parameter space can be guided by the baseline neural activity in the target areas. A way to estimate the nonlinear functions of how the latent state evolves over time, and the relationship between the latent state and observed signals, is to treat the functions as random objects whose priors are Gaussian processes (Wang et al., 2008). These functions can be learned by applying the Bayesian methodology as described in section “7. Leveraging machine learning”.

Accept Letter

Dear Prof Saalmann,

Thank you for submitting your manuscript to Current Research in Neurobiology.

I am pleased to inform you that your manuscript has been accepted for publication. Congratulations!

My comments, and any reviewer comments, are below.

Your accepted manuscript will now be transferred to our production department. We will create a proof

which you will be asked to check, and you will also be asked to complete a number of online forms required for publication. If we need additional information from you during the production process, we will contact you directly.

We appreciate and value your contribution to Current Research in Neurobiology. We regularly invite authors of recently published manuscript to participate in the peer review process. If you were not already part of the journal's reviewer pool, you have now been added to it. We look forward to your continued participation in our journal, and we hope you will consider us again for future submissions.

CRNEUR aims to be a unique, community-led journal, as highlighted in the Editorial Introduction. As part of this vision, we will be regularly seeking input from the scientific community and encourage you and your co-authors to take the survey.

We would also like to invite you to take part in our CRNEUR Author Question & Answer (Q&A), which could get published alongside your article and help to promote it. We suspect you might have an interesting story of perseverance or team work that was required for the research study to complete, or a diversity of perspectives that you might share, as a way of inspiring others about neuroscience.

Kind regards,

Anna S Mitchell, Ph.D.
Editor in Chief
Current Research in Neurobiology

Editor and Reviewer comments:

I very much enjoyed reading this revised manuscript - Well done.

Reviewer #2: I must congratulate the authors for their revision and their efforts. This improved significantly their manuscript.
I have no additional concerns to raise. This new concept is very exciting although very challenging to set up in the clinics.

-------- *End of Review Comments* --------
